# Monocyte chemotactic protein-1 deficiency reduces spontaneous metastasis of Lewis lung carcinoma in mice fed a high-fat diet

**DOI:** 10.18632/oncotarget.8364

**Published:** 2016-03-25

**Authors:** Lin Yan, Sneha Sundaram

**Affiliations:** ^1^ U.S. Department of Agriculture, Agricultural Research Service, Grand Forks Human Nutrition Research Center, Grand Forks, ND 58202, U.S.A.

**Keywords:** MCP-1, pro-inflammatory cytokine, metastasis, high-fat diet, mice

## Abstract

Adipose-produced pro-inflammatory cytokines contribute to obesity and cancer. This 2×2 experiment was designed to investigate effects of monocyte chemotactic protein-1 (MCP-1) deficiency on pulmonary metastasis of Lewis lung carcinoma (LLC) in MCP-1 deficient and wild-type mice fed a modified AIN93G diet containing 16% and 45% of energy from corn oil, respectively. The high-fat diet significantly increased the number and size (cross-sectional area and volume) of lung metastases compared to the AIN93G control diet. Deficiency in MCP-1 reduced lung metastases by 37% in high-fat diet-fed mice; it reduced metastatic cross-sectional area by 46% and volume by 69% compared to wild-type mice. Adipose and plasma concentrations of MCP-1 were significantly higher in high-fat diet-fed wild-type mice than in their AIN93G-fed counterparts; they were not detectable in MCP-1 deficient mice regardless of diet. Plasma concentrations of plasminogen activator inhibitor-1, tumor necrosis factor-α, vascular endothelial growth factor and tissue inhibitor of metalloproteinase-1 were significantly higher in MCP-1 deficient mice compared to wild-type mice. We conclude that adipose-produced MCP-1 contributes to high-fat diet-enhanced metastasis. While MCP-1 deficiency reduces metastasis, the elevation of pro-inflammatory cytokines and angiogenic factors in the absence of MCP-1 may support the metastatic development and growth of LLC in MCP-1 deficient mice.

## INTRODUCTION

Monocyte chemotactic protein-1 (MCP-1) is a member of the chemokine family composed of 76 amino acids, and it is 13 kDa in size [1]. Monocyte chemotactic protein-1 is primarily identified as a potent chemotactic factor for attracting monocytes, macrophages and other inflammatory cells to the site of inflammation during tissue injury and infection [2]. Studies in recent years reveal that functions of MCP-1 are far beyond tissue repair; it participates in pathophysiological development of various diseases including cancer and obesity.

High levels of MCP-1 are associated with a poor outcome and a short disease-free survival after surgery because of the high incidence of metastasis in patients with cancer [3–5]. For example, high expression of MCP-1 is positively associated with advanced pathologic stages of prostate cancer [3] and with multiple liver metastases of colorectal cancer [5]. In breast cancer, it is a significant indicator of early relapse [4]. Animal studies indicate that MCP-1 participates in primary tumor growth and metastasis [6–8]. Treatment of mice with a neutralizing antibody to MCP-1 reduces the growth and metastasis of non-small-cell lung carcinoma [6]; stable knockdown of MCP-1 in MDA-MB-231 mammary carcinoma cells reduces their metastasis *in vivo* [7].

Obesity is a risk factor for cancer. Accumulation of adipose tissue in viscera is a strong indicator of detrimental health outcomes in obesity-associated diseases including cancer [9, 10]. A variety of cell types (e.g. hepatocytes, fibroblasts) produce MCP-1 [11]; among them adipocytes are recognized as an important source of MCP-1 [9, 10]. Adipose MCP-1 mRNA expression is correlated with adiposity and body mass index, and circulating MCP-1 is reduced after weight loss in obese subjects [12]. Being obese at the time of diagnosis of primary cancer can be predictive of poor prognosis. For example, breast cancer patients who are obese are at a greater risk of recurrence [13] with a shorter disease-free interval than those with normal body weight [14]. Similarly, obese or overweight prostate cancer patients are more likely to have prostate cancer recurrence after radical prostatectomy than those of normal weight [15, 16]. These observations suggest that pro-inflammatory adipokines may interact with cancer cells and play an active role in obesity-associated cancer progression.

Feeding laboratory rodents an obesogenic, high-fat diet results in increases in body fat mass and plasma concentration of MCP-1 [17–19]. In our studies on the roles of diet in metastasis using the spontaneous metastasis model of Lewis lung carcinoma (LLC), we found that high-fat diet enhances metastasis, which is accompanied by increases in plasma concentrations of adipokines including MCP-1 [20, 21]. We hypothesized that MCP-1 participates in metastatic development and growth and that adipose-produced MCP-1 contributes to the high-fat diet-enhanced metastasis. The present experiments were conducted to test the hypothesis in MCP-1 deficient (MCP-1^−/−^) mice using the spontaneous metastasis model of LLC.

## RESULTS

**Figure 1:**
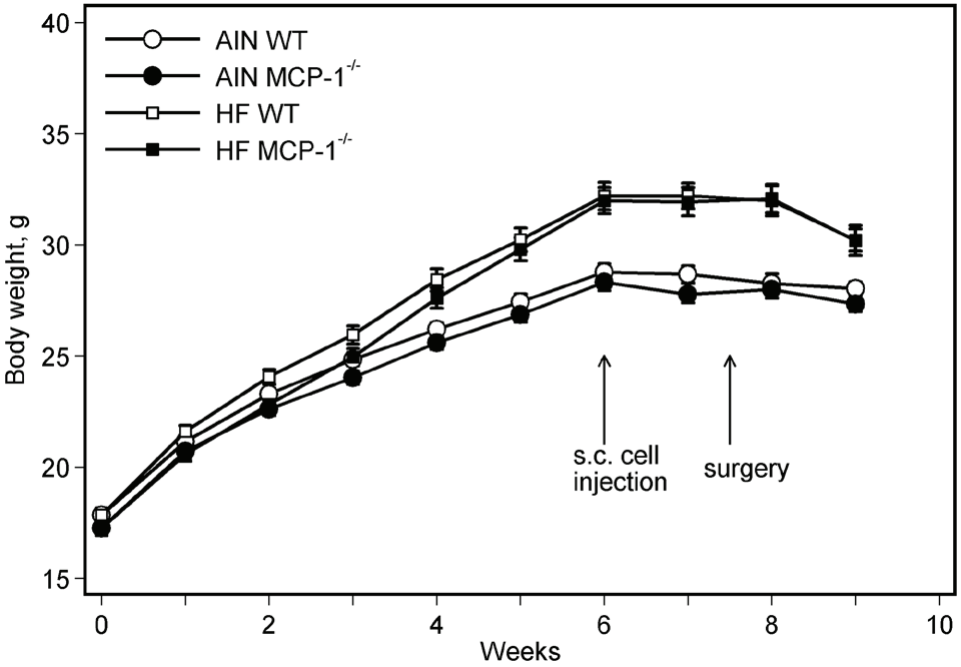
Body weight Two-way ANOVA and Tukey contrasts were performed to test for differences among the groups. Mice fed the high-fat diet were heavier than those fed the AIN93G diet; the difference was significant starting 3 weeks after initiation of experimental feeding (*p* < 0.01). Body weights of MCP-1^−/−^ mice were lower than those of wild-type mice during the first 3 weeks of experimental feeding (*p* < 0.05); there were no differences in body weights between MCP-1^−/−^ and wild-type mice receiving the same dietary treatment thereafter. Values are means ± SEM (n = 21 per group for MCP-1^−/−^ mice, n = 28 per group for wild-type mice). AIN: AIN93G diet; WT: wild-type mice; MCP-1^−/−^: MCP-1 deficient mice; HF: high-fat diet.

Consumption of the high-fat diet, regardless of genotypes of mice, increased body weight compared to the AIN93G diet. The difference was statistically significant 3 weeks after initiation of experimental feeding (*p* < 0.01), and the significant increase continued throughout the experiment (Figure 1). Body weights of MCP-1^−/−^ mice were lower than those of wild-type mice during the first 3 weeks of experimental feeding (*p* < 0.05); there were no differences in body weights between MCP-1^−/−^ and wild-type mice receiving the same dietary treatment thereafter (Figure 1).

**Figure 2:**
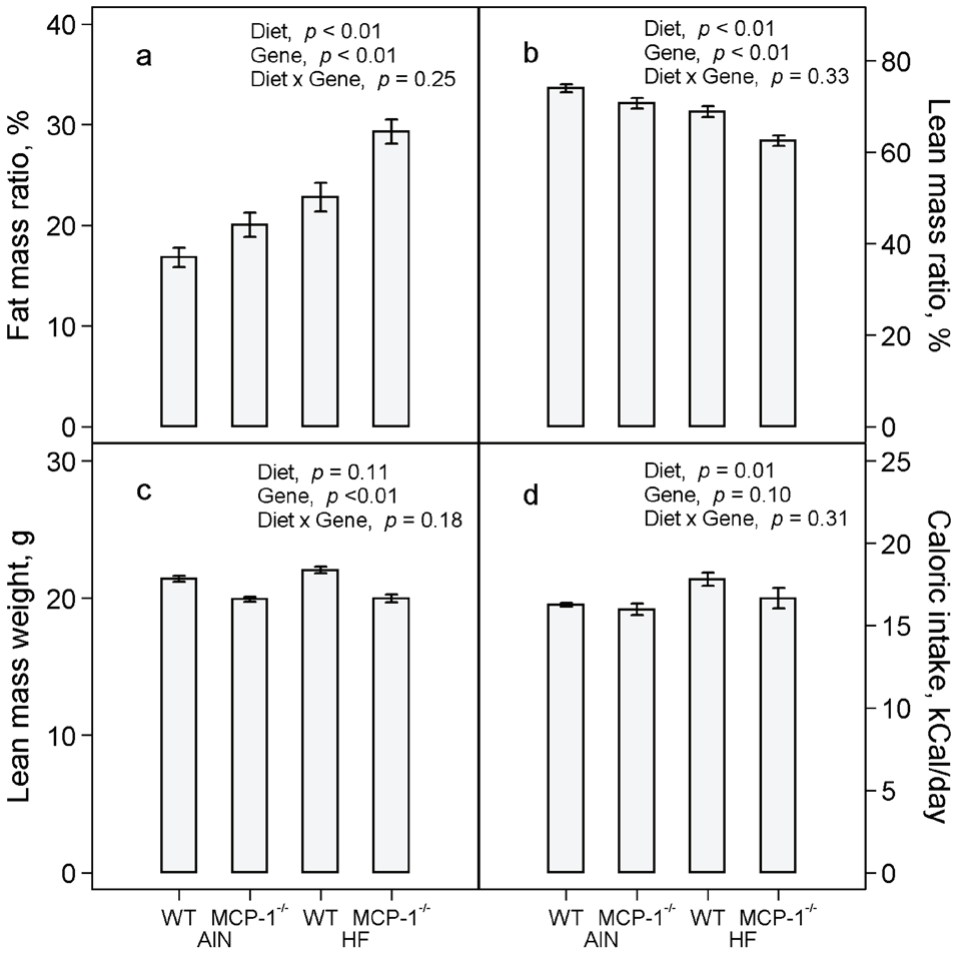
Fat mass:body mass ratio **a.** lean mass:body mass ratio **b.** lean mass weight **c.** and caloric intake **d.** Two-way ANOVA was performed to test for differences among the groups. Values are means ± SEM (n = 21 per group for MCP-1^−/−^ mice, n = 28 per group for wild-type mice (a,b,c) or n = 6 per group (d)). AIN: AIN93G diet; WT: wild-type mice; MCP-1^−/−^: MCP-1 deficient mice; HF: high-fat diet.

The high-fat diet increased percent body fat mass by 44% (*p* < 0.01) compared to the AIN93G diet; MCP-1 deficiency increased body fat mass by 27% (*p* < 0.01) compared to wild-type mice (Figure 2a). Concomitantly, the high-fat diet reduced percent body lean mass by 10% (*p* < 0.01) and MCP-1 deficiency reduced it by 7% (*p* < 0.01) compared to their respective controls (Figure 2b). There was no difference in absolute lean mass weight between groups fed the high-fat and the AIN93G diets (Figure 2c); lean mass weights of MCP-1^−/−^ mice were 8% lower than those of wild-type mice (*p* < 0.01, Figure 2c). Consumption of the high-fat diet increased caloric intake by 7% (*p* = 0.01) compared to the AIN93G diet; there was no difference in caloric intake between MCP-1^−/−^ and wild-type mice (Figure 2d).

**Figure 3:**
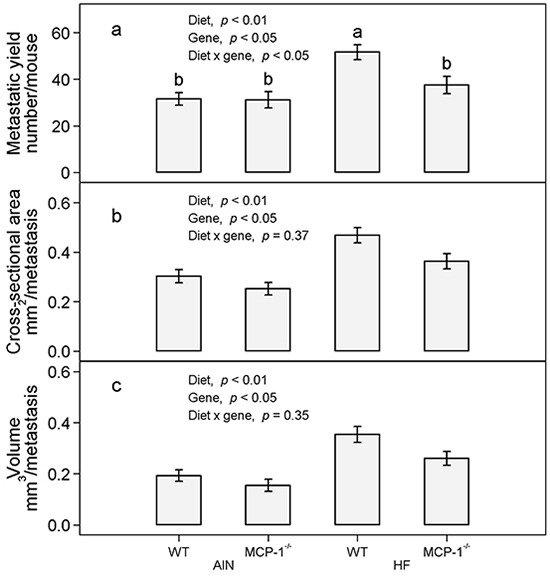
The number **a.** cross-sectional area **b.** and volume **c.** of pulmonary metastases in MCP-1^−/−^ and wild-type mice fed the AIN93G or the high-fat diet. Two-way ANOVA and Tukey contrasts were performed to test for differences among the groups. Values (means ± SEM) with different superscripts are significantly different at *p* < 0.05 (n = 21 per group for MCP-1^−/−^ mice, n = 28 per group for wild-type mice). AIN: AIN93G diet; WT: wild-type mice; MCP-1^−/−^: MCP-1 deficient mice; HF: high-fat diet.

Subcutaneous injection of LLC cells resulted in a primary tumor at the injection site and pulmonary metastasis. The high-fat diet increased the number of lung metastases in wild-type mice by 64% compared to their AIN93G-fed counterparts (*p* < 0.05, Figure 3a). Deficiency in MCP-1 reduced the number of metastases in high-fat diet-fed mice by 27% (*p* < 0.05), but it did not affect the number of metastases in AIN93G-fed mice (Figure 3a). Compared to the AIN93G diet, the high-fat diet increased the cross-sectional area and volume of pulmonary metastases by 46% (*p* < 0.01, Figure 3b) and 69% (*p* < 0.01, Figure 3c), respectively. Compared to wild-type mice, deficiency in MCP-1 reduced the cross-sectional area and volume of metastases by 17% (*p* < 0.05, Figure 3b) and 21% (*p* < 0.05, Figure 3c), respectively.

**Figure 4:**
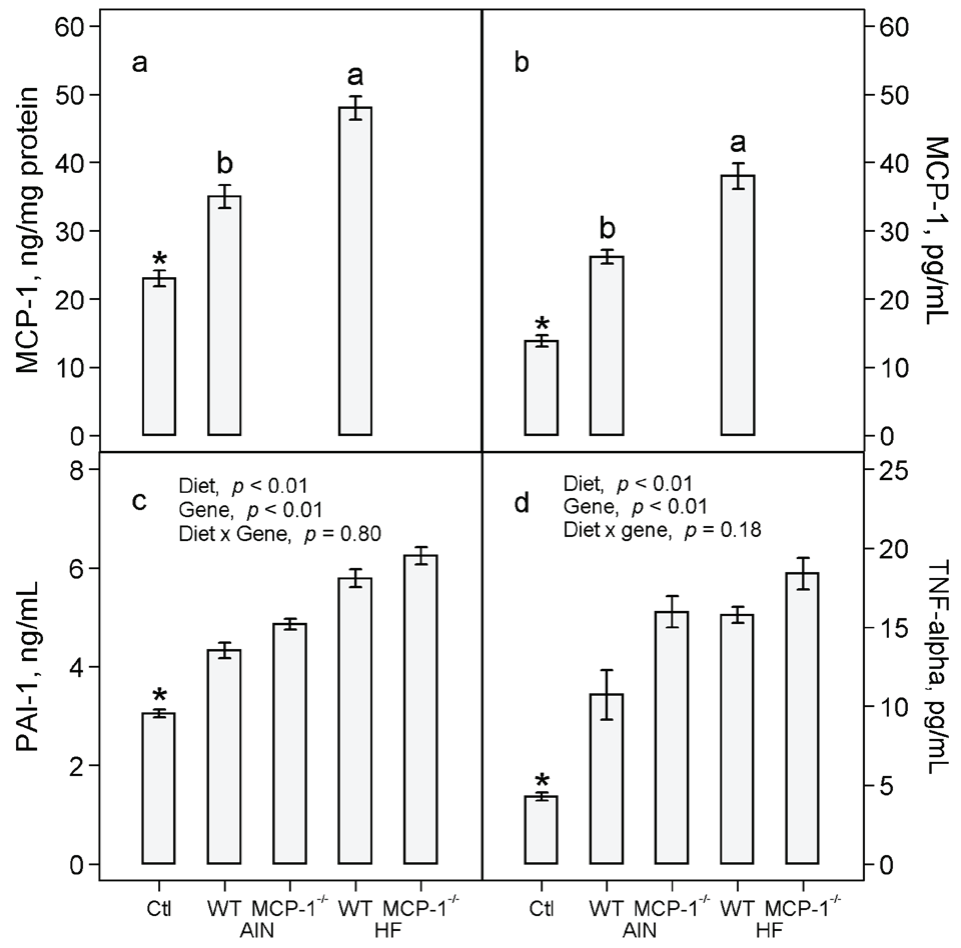
Concentrations of MCP-1 in adipose tissue **a.** and plasma **b.** and concentrations of PAI-1 **c.** and TNF-α **d.** in plasma. Two-way ANOVA and Tukey contrasts were performed to compare differences among the groups of LLC-bearing mice; *a priori* contrasts were performed to compare differences in mice fed the AIN93G diet with or without LLC. Values (means ± SEM) with different superscripts are significantly different at *p* ≤ 0.05 for LLC-bearing groups (n = 10 per group). *p < 0.01 compared to AIN WT. Ctl: non-tumor-bearing wild-type mice fed the AIN93G diet; AIN: AIN93G diet; WT: wild-type mice; MCP-1^−/−^: MCP-1 deficient mice; HF: high-fat diet.

In wild-type mice fed the AIN93G diet, LLC increased concentrations of MCP-1 in adipose tissue by 52% (*p* < 0.01, Figure 4a) and plasma by 89% (*p* < 0.01, Figure 4b) compared to non-tumor-bearing controls. The high-fat diet further increased concentrations of MCP-1 in adipose tissue by 37% (*p* < 0.01, Figure 4a) and in plasma by 45% (*p* < 0.05, Figure 4b) in LLC-bearing mice compared to the AIN93G diet. Monocyte chemotactic protein-1 was not detectable in adipose tissue and plasma of MCP-1^−/−^ mice regardless of diet (Figure 4a and 4b). The concentration of MCP-1 in 24-hour-old serum-free conditioned medium from LLC cells was 2.0 ± 0.1 ng/mg protein (n = 10). Concentrations of MCP-1 in primary tumors of MCP-1^−/−^ and wild-type mice were 235.0 ± 10.1 and 217.0 ± 10.1 pg/mg protein (*p* = 0.21, n = 20), respectively.

Lewis lung carcinoma increased plasma concentrations of PAI-1 by 42% (*p* < 0.01, Figure 4c) and TNF-α by 2.5 fold (*p* < 0.01, Figure 4d) in wild-type mice compared to non-tumor-bearing controls fed the AIN93G diet. The high-fat diet increased plasma PAI-1 by 31% (*p* < 0.01, Figure 4c) and TNF-α by 28% (*p* < 0.01, Figure 4d) in LLC-bearing mice compared to the AIN93G diet. Deficiency in MCP-1 increased PAI-1 by 10% (*p* < 0.01, Figure 4c) and TNF-α by 30% (*p* < 0.01, Figure 4d) compared to wild-type mice.

**Figure 5:**
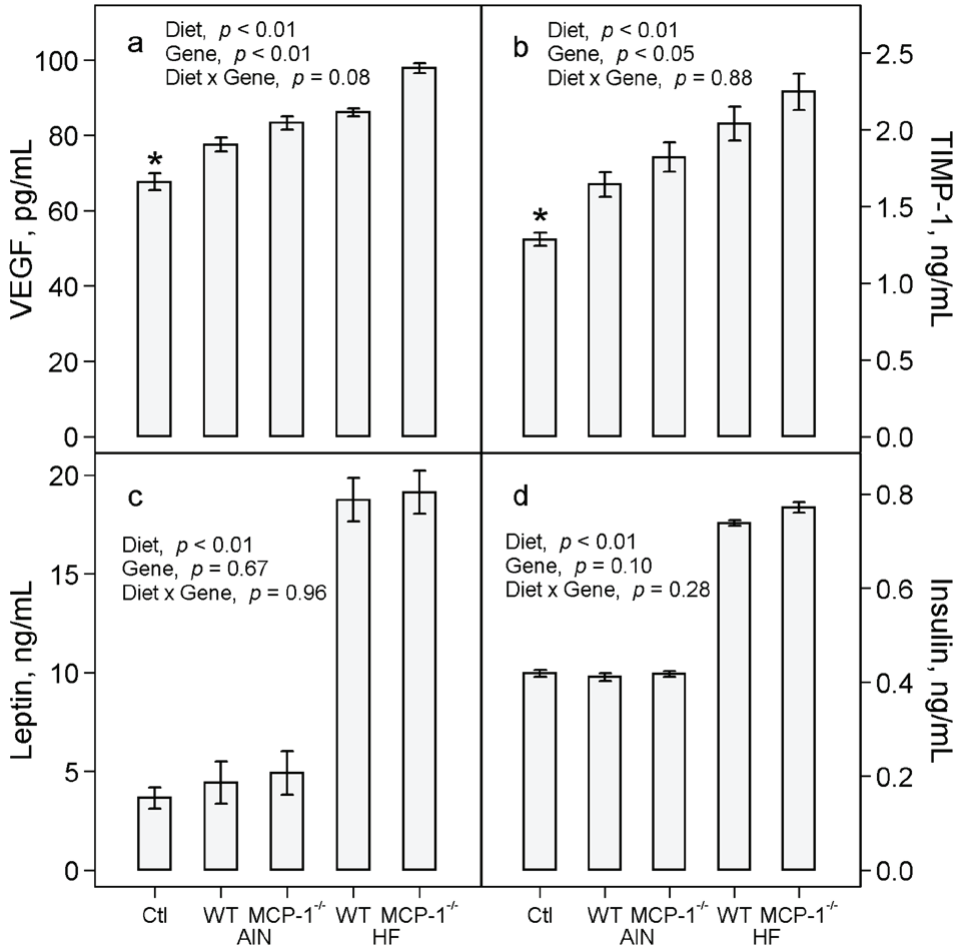
Plasma concentrations of VEGF **a.** TIMP-1 **b.** leptin **c.** and insulin **d.** Two-way ANOVA was performed to compare differences among the groups of LLC-bearing mice; *a priori* contrasts were performed to compare differences in mice fed the AIN93G diet with or without LLC. Values are means ± SEM (n = 10 per group). *p ≤ 0.01 compared to AIN WT. Ctl: non-tumor-bearing wild-type mice fed the AIN93G diet; AIN: AIN93G diet; WT: wild-type mice; MCP-1^−/−^: MCP-1 deficient mice; HF: high-fat diet.

Mice with LLC exhibited a 15% increase in plasma concentration of VEGF (*p* < 0.01, Figure 5a) and a 28% increase in TIMP-1 (*p* = 0.01, Figure 5b) compared to non-tumor-bearing controls. The high-fat diet increased plasma VEGF by 14% (*p* < 0.01, Figure 5a) and TIMP-1 by 24% (*p* < 0.01, Figure 5b) in LLC-bearing mice compared to the AIN93G diet. Deficiency in MCP-1 increased plasma VEGF by 11% (*p* < 0.01, Figure 5a) and TIMP-1 by 11% (*p* < 0.05, Figure 5b) compared to wild-type mice.

There were no differences in plasma concentrations of leptin and insulin between LLC-bearing and non-tumor-bearing wild-type mice (Figure 5c and 5d). Consumption of the high-fat diet resulted in a 4-fold increase in plasma leptin (*p* < 0.01, Figure 5c) and an 82% increase in plasma insulin (*p* < 0.01, Figure 5d) compared to the AIN93G diet. Deficiency in MCP-1 did not affect plasma concentrations of either compared to wild-type mice (Figure 5c and 5d).

## DISCUSSION

Results from the present study are consistent with previous reports that feeding mice an obesogenic, high-fat diet elevates plasma concentration of MCP-1 [18, 19] and enhances pulmonary metastasis [20, 22]. The key finding is that deficiency in MCP-1 reduces high-fat diet-enhanced metastasis, reflected by reductions in both number and size of metastases formed in lungs (Figure 3). It indicates that MCP-1 plays an active role in promoting metastatic progression and that adipose-produced MCP-1 may contribute, at least partly, to high-fat diet-enhanced metastasis. These findings are supported by previous studies showing that treatment of mice with a neutralizing antibody to MCP-1 reduces the growth and metastasis of non-small-cell lung carcinoma [6].

Deficiency in MCP-1 resulted in significant elevations of plasma concentrations of pro-inflammatory cytokines (PAI-1, TNF-α) and angiogenic factors (VEGF, TIMP-1) in LLC-bearing mice (Figures 4 and 5). These elevations apparently compensate for the absence of MCP-1. Plasminogen activator inhibitor-1 promotes metastasis [22, 23] and malignant growth [22, 24]. Tumor necrosis factor-α [25, 26], VEGF [27, 28] and TIMP-1 [29] are potent tumorigenic and angiogenic factors that contribute to cancer progression. Thus, the observed elevations of pro-inflammatory cytokines and angiogenic factors may offset the absence of MCP-1, thereby supporting the metastatic progression of LLC in MCP-1^−/−^ mice.

The mechanisms by which MCP-1 deficiency increases plasma concentrations of pro-inflammatory cytokines and angiogenic factor remain to be elucidated. Available studies show that TNF-α induces the expression of MCP-1 in cancer-associated fibroblasts and mesenchymal stem cells by up-regulating the nuclear factor-κB (NF- κB) pathway [30]. The elevation in plasma concentration of TNF-α in the absence of MCP-1 may be due to a feedback response via the NF-κB/TNF-α pathway.

Concentrations of the adipokine MCP-1 in adipose tissue were elevated in LLC-bearing wild-type mice fed the AIN93G diet (Figure 4). Elevated expression of adipokines during interaction between adipocytes and cancer cells contributes to cancer progression. For example, concentration of MCP-1 is greater in cancer-associated adipocytes from human mastectomy samples than normal adipocytes [31], and MCP-1 expression is higher in peritumoral adipose tissue than control adipose tissue in mice [32]. The LLC cell line used in this study is a variant that metastasizes specifically to lungs [33]. Thus, the observed elevation of MCP-1 in adipose tissue is unlikely a result of interactions between cancer cells and adipocytes but may be through mechanisms related to other aspects of metastasis. This warrants further investigation.

Consistent with our previous reports [18, 22], feeding mice the high-fat diet elevated concentrations of leptin and insulin in plasma (Figure 5). The lack of differences in these measurements between MCP-1^−/−^ and wild-type mice indicates that the observed elevations are independent from MCP-1 status of the mice. Elevations of leptin and insulin contribute to obesity in rodent models [18, 34]. Leptin is angiogenic during tumorigenesis [35] and insulin is involved in type-2 diabetes-mediated mammary tumor progression [36] in mice. Thus, the present results raise the possibility that elevations of leptin and insulin may contribute to the high-fat diet-enhanced metastasis.

The finding that MCP-1^−/−^ mice gained more body fat mass than their wild-type counterparts (Figure 2) was unexpected. However, these results are supported by previous reports that MCP-1^−/−^ mice fed a high-fat diet gain more body fat mass than wild-type mice [37] and that obese MCP-1^−/−^ mice have larger adipocytes [38]. Taken together, these results indicate that MCP-1 deficiency does not attenuate diet-induced adipogenesis but increases body fat mass build-up in this rodent model.

It is generally accepted that it is the fat content of a diet that is responsible for the increases in body adiposity and its associated adipokines. Studies of obesity in mouse models commonly use 45% of energy from fat in diets. We recently reported that restricted feeding of a high-fat diet during the dark cycle (12 hours) does not affect energy intake but reduces body fat mass and plasma concentration of MCP-1 compared to mice fed the same diet *ad libitum* [39]. It suggests that it is not the fat content of a diet but the disruption of diurnal rhythm of food intake that may be responsible for adipogenesis in mice. This needs to be tested on high-fat diet-enhanced metastasis.

In summary, results from this study showed that MCP-1 deficiency ameliorates metastasis in high-fat diet-fed mice indicating that adipose-produced MCP-1 contributes, at least partly, to high-fat diet-enhanced metastasis. Furthermore, results that plasma concentrations of pro-inflammatory cytokines and angiogenic factors were elevated in MCP-1^−/−^ mice indicates the existence of compensatory mechanisms that up regulates the production of these cancer promoting factors, which support LLC progression in the absence of MCP-1. These results suggest that inhibition of MCP-1 expression, either by preventive or therapeutic approaches, may attenuate metastatic progression. Elevations in plasma levels of pro-inflammatory cytokines and angiogenic factors in the absence of MCP-1 suggest the existence of compensatory responses that may counteract treatments aimed at MCP-1 inhibition.

## MATERIALS AND METHODS

### Animals and diets

Four to five-week-old male MCP-1 deficient mice (MCP-1^−/−^, B6.129S4-*Ccl2^tm1Rol^*/J) on a C57BL/6J background and C57BL/6J wild-type mice (The Jackson Laboratory, Bar Harbor, ME) were maintained in a pathogen-free room on a 12:12-hour light/dark cycle with a temperature of 22 ± 1°C. A modified AIN93G formulation [40] providing 16% or 45% (high-fat diet) of energy from corn oil was used in this study (Table 1). Gross energy of each diet (Table 1) was analyzed by oxygen bomb calorimetry (Model 6200, Oxygen Bomb Calorimeter, Parr Instrument, Moline, IL). All diets were powder diets; they were stored at −20°C until being provided to mice. Mice had free access to their diets and deionized water; food intake was recorded for 3 weeks before injection of cancer cells. Mice were weighed weekly; body composition was assessed in conscious, immobilized mice using quantitative magnetic resonance imaging 1 week before the cancer cell injection (Echo whole-body composition analyzer, Model 100, Echo Medical System, Houston, TX).

**Table 1:** Composition of experimental diets

Ingredient	Modified AIN93G g/kg	High-fat g/kg
Corn starch	390	34
Casein	200	239
Dextrin	132	239
Sucrose	100	120
Corn oil	70	229
Cellulose	50	60
AIN93 mineral mix	35	42
AIN93 vitamin mix	10	12
L-cystine	4.4	5
L-methionine	0.3	0.4
Choline bitartrate	2.5	3
Sodium carbonate	6.22	7
*t*-Butylhydroquinone	0.01	0.05
Total	1000	1000
***Energy***	***%***	***%***
Protein	20	20
Fat	16	45
Carbohydrate	64	35
Gross energy^a^, kCal/g	4.4 ± 0.1	5.4 ± 0.1

a n =3 for each diet.

### Lewis lung carcinoma cells

Lewis lung carcinoma cells (provided by Dr. Pnina Brodt, McGill University, Montreal, Quebec, Canada) were cultured with RPMI-1640 medium containing 10% heat-inactivated fetal bovine serum and maintained in a humidified atmosphere of 5% CO_2_ in air at 37°C. Cells used for animal studies were *in vivo*-selected once [21]; they were monitored for phenotype (by microscopic examination of cell morphology), proliferation properties (by growth curve analysis) and metastatic capability (by injecting cells subcutaneously into mice and examining metastatic formation in lungs). Cells were free of mycoplasma by Hoechst DNA staining and direct culture tests (performed by the American Type Culture Collection, Manassas, VA). Such assessments showed that cell identity and metastatic behavior were similar to those of original stocks from the institution providing the cell line.

### Experimental design

This study was approved by the Animal Care and Use Committee of the U.S. Department of Agriculture, Agricultural Research Service, Grand Forks Human Nutrition Research Center. The procedures followed the National Institutes of Health guidelines for the care and use of laboratory animals [41]. Mice (n = 21 per group for MCP-1^−/−^ mice, n = 28 per group for wild-type mice) were fed the AIN93G control or high-fat diet for 7 weeks before they were subcutaneously injected with 2.5 × 10^5^ viable LLC cells per mouse into the lower dorsal region. The resulting primary tumor was removed surgically 11 days later when it was approximately 1 cm in diameter; after that mice were maintained on their respective diets for an additional 10 days. Mice fed the AIN93G diet but not injected with LLC cells were used as non-tumor-bearing controls (n = 18) in comparison of plasma concentrations of pro-inflammatory cytokines, angiogenic factors and insulin to LLC-bearing mice fed the same AIN93G diet. At termination, mice were euthanized with an intraperitoneal injection of a mixture of ketamine and xylazine; their lungs were harvested and fixed with Bouin's solution. The number of pulmonary metastases was counted and the cross-sectional area and the average diameter of each metastasis were measured using an ImagePro-Plus software- (Media Cybernetics, Silver Spring, MD) and camera-equipped stereomicroscope. The cross-sectional area was defined as the surface area of each lung metastasis. The average diameter was measured at two degree intervals joining two outline points and passing through the centroid. The volume of each metastasis was estimated with the assumption that metastases were spherical and calculated using the average diameter measured [42]. Epididymal adipose tissue and plasma were collected and stored at −80°C for further analyses.

### Quantification of cytokines, angiogenic factors and related biomarkers

Concentrations of MCP-1 in epididymal adipose tissue, plasma, 24-hours-old serum-free conditioned medium from LLC cells and primary tumors were quantified by using a sandwich enzyme-linked immunosorbent assay (ELISA) kit (R&D Systems, Minneapolis, MN). Primary tumors frozen in liquid nitrogen were pulverized under liquid nitrogen. Total protein from adipose tissue and primary tumors was extracted in RIPA buffer with protease and phosphatase inhibitors [43]. Protein concentrations of the adipose and primary tumor extract and LLC cell lysate were estimated by using the BCA protein assay (Thermo Scientific, Waltham, MA). Concentrations of MCP-1 in adipose tissue, primary tumor and conditioned medium were normalized to protein content. Concentrations of plasminogen activator inhibitor-1 (PAI-1, Molecular Innovations, Inc., Novi, MI), insulin (Mercodia, Inc., Winston Salem, NC), tumor necrosis factor-α (TNF-α), leptin, vascular endothelial growth factor (VEGF) and tissue inhibitor of metalloproteinase-1 (TIMP-1, R&D Systems, Minneapolis, MN) in plasma were quantified by using ELISA kits following manufacturers' protocols. Samples were read within the linear range of the assay, and the accuracy of the analysis was confirmed with the controls provided in each kit.

### Statistical analyses

The effects of diet (AIN93G or high-fat), genotype (MCP-1^−/−^ or wild-type) and their interaction were tested by using two-way analysis of variance (ANOVA). In case of a significant interaction between diet and genotype, Tukey contrasts were performed to compare the 4 dietary groups. To examine the effect of LLC on plasma concentrations of cytokines, angiogenic factors and insulin, *a priori* contrasts were used to test for differences in wild-type mice fed the AIN93G diet with or without LLC. A mixed model ANOVA with mouse as the random blocking factor and with diet, genotype and their interaction as fixed effects was used to test for differences in cross-sectional area and volume of metastases among the groups. All data are presented as means ± standard error of the mean (SEM). Differences with a *p*-value of 0.05 or less were considered statistically significant. All analyses were performed by using SAS software (version 9.4, SAS Institute, Cary, NC).
